# Three-dimensional computed tomography reconstruction in video-assisted thoracoscopic segmentectomy (DRIVATS): A prospective, multicenter randomized controlled trial

**DOI:** 10.3389/fsurg.2022.941582

**Published:** 2022-10-13

**Authors:** Zhenyi Niu, Kai Chen, Runsen Jin, Bin Zheng, Xian Gong, Qiang Nie, Benyuan Jiang, Wenzhao Zhong, Chun Chen, Hecheng Li

**Affiliations:** ^1^ Department of Thoracic Surgery, Ruijin Hospital, Shanghai Jiao Tong University School of Medicine, Shanghai, China; ^2^ Department of Thoracic Surgery, Fujian Medical University Fujian Union Hospital, Fuzhou, China; ^3^ Guangdong Lung Cancer Institute, Guangdong Provincial Key Laboratory of Translational Medicine in Lung Cancer, Guangdong Provincial People’s Hospital / Guangdong Academy of Medical Sciences, Guangzhou, China

**Keywords:** 3D reconstruction CT, segmentectomy, video-assisted thoracoscopic, pulmonary nodules, multicenter randomized controlled trial

## Abstract

**Objective:**

Anatomical segmentectomy has been proven to be a viable surgical treatment for small-size peripheral lung nodules. Three-dimensional (3D) reconstruction computed tomography (CT) has been proposed as an effective approach to overcome the challenges of encountering pulmonary anatomical variations when performing segmentectomy. Therefore, to further investigate the usefulness of preoperative 3D reconstruction CT in segmentectomy, we will conduct this prospective, multicenter randomized controlled DRIVATS study to compare the use of 3D reconstruction CT with standard chest CT in video-assisted segmentectomy (ClinicalTrials.gov ID: NCT04004494).

**Methods:**

This study began in July 2019 and a total of 190 patients will be accrued from three clinical centers within 4 years. The main inclusion criteria are patients with a single peripheral nodule 0.8–2 cm with at least one of the following requirements: (i) histology of adenocarcinoma *in situ*; (ii) nodule has ≥50% ground-glass appearance on CT; (iii) radiologic surveillance confirms a long doubling time (≥400 days). Surgical procedures include segmental resection of the lesion and mediastinal lymph node sampling (subsegmental resection or combined subsegmental resection will not be included in this study). The primary endpoint is operative time. The secondary endpoints include incidence of change of surgical plan, intraoperative blood loss, conversion rate, operative accident event, incidence of postoperative complications, postoperative hospital stay, length of hospitalization, duration of chest tube placement, postoperative 30-day mortality, dissection of lymph nodes, overall survival, disease-free survival, preoperative lung function, and postoperative lung function.

**Discussion:**

This multicenter DRIVATS study aims to verify the usefulness of preoperative 3D reconstruction CT compared with standard chest CT in segmentectomy. If successfully completed, this multicenter prospective study will provide a higher level of evidence for the use of 3D reconstruction CT in segmentectomy.

## Introduction

Lung cancer is the leading cause of cancer-related death across the world (1). In 2020, lung cancer remained the leading cause of cancer death, with an estimated 1.8 million deaths (2). Non-small-cell lung cancer (NSCLC) is the predominant type of lung cancer, which takes up 85% of lung cancer (3). With the increasing usage of new screening technology such as low-dose computed tomography (CT), the detection of small pulmonary nodules, particularly small ground-glass opacity (GGO) nodules, has increased (4). There is an increasing interest in pursuing anatomical segmentectomy for these presumably low-risk tumors, especially for patients with impaired pulmonary function reserve. A series of studies conducted have validated the feasibility of segmentectomy in the treatment of these small-size nodules, which could achieve a long-term oncological effect comparable to that achieved with standard lobectomy (5, 6).

Maintaining an accurate surgical plane and a secure surgical margin is of great importance for a successful segmentectomy of a small and relatively deep pulmonary nodule, which makes it more challenging than lobectomy and identifies the need for a thorough understanding of the segmental anatomy. Vascular and bronchial variations of lung segments are inevitable. Anatomical variations of pulmonary vessels can cause serious problems such as unwanted bleeding in patients undergoing video-assisted thoracic surgery (VATS) (7–9) and some variation patterns of pulmonary vessels have been summarized in previous studies (10–13). Careful preoperative evaluation and surgical techniques are needed to prevent intraoperative damage due to unexpected anatomic anomalies. The technique of three-dimensional (3D) reconstruction CT has received a lot of attention as it can provide accurate information about the nodule segmental location as well as its relationship with intersegmental lines (14).

Some new 3D imaging software programs using 2D CT data have been developed and have shown some advantages in planning accurate segmental resections (15–18). A series of retrospective studies have been conducted, which have shown that this 3D reconstruction CT technique has some benefits in a clear delineation of the anatomy of the pulmonary vessel branches and adequate identification of anatomic variations (17, 19–22). Compared to patients who received preoperative standard chest CT, significantly shorter operative time and less intraoperative blood loss were observed in patients receiving preoperative 3D reconstruction CT (22, 23). Most of the studies that evaluated the benefits of preoperative 3D reconstruction CT were retrospective studies, with only one quasi-randomized clinical trial (23). However, in this quasi-randomized clinical trial, the number of patients included in the two groups was limited. Taken together, these results highlight the need for further investigation with preoperative 3D reconstruction CT in segmentectomy.

The results of previous studies justify a further prospective evaluation of the use of preoperative 3D reconstruction CT in segmental resections. Therefore, we designed and conducted this multicenter, randomized controlled DRIVATS study to compare the usefulness of 3D reconstruction CT and standard chest CT in preoperative planning of video-assisted segmentectomy (ClinicalTrials.gov ID: NCT04004494).

## Materials and methods

### Study design

The DRIVATS study is a prospective, multicenter, randomized controlled clinical trial to compare the usefulness of 3D reconstruction CT and standard chest CT in preoperative planning of video-assisted segmentectomy. Three high-volume medical centers in China are participating in this study (Ruijin Hospital, Shanghai Jiao Tong University School of Medicine; Fujian Medical University Union Hospital; and Guangdong Provincial People's Hospital). Ethics approval has been obtained from Ruijin Hospital Ethics Committee on March 21, 2019 (approval number, 2019-19). Other two clinical centers have also obtained ethics approval from their local ethics committee (approval number, 2019YF032-01 and GDREC2019522H) and a copy has been forwarded to the Central Coordinating Centre. All patients are required to sign an informed consent form after personal counseling by an independent research coordinator. This study was initiated in July 2019. With an estimated inclusion period of 4 years, the primary endpoint is anticipated to be achieved in December 2023.

### Eligibility criteria

The inclusion criteria are as follows:

• Age older than 18 years.
• Pulmonary nodules or GGO found in chest CT examination and conform with indications for segmentectomy:
• Peripheral nodule 0.8–2 cm with at least one of the following requirements:
  I. Histology of adenocarcinoma *in situ*,
  II. Nodule has ≥50% ground-glass appearance on CT, and
  III. Radiologic surveillance confirms a long doubling time (≥400 days).
• Adequate cardiac function, respiratory function, liver function, and renal function for anesthesia and VATS segmentectomy.
• American Society of Anesthesiologists (ASA) score: Grade I–III.
• Patients who can coordinate the treatment and research and sign the informed consent.

The exclusion criteria are as follows:

• Patients with significant medical condition which is thought unlikely to tolerate the surgery. For example, cardiac disease, and significant liver and renal function disorder.
• Patients with psychiatric diseases who may not comply with the protocol.
• Patients with a history of chest trauma or surgery on the ipsilateral chest, which may cause pleural adhesion.

### Endpoints

The primary endpoint of this study is operative time.

The secondary endpoints are listed as follows:

• Incidence of change of surgical plan: change of surgical plan will be recorded when the actual resected bronchus and vessels are different from those decided in the preoperative surgical plan;
• Intraoperative blood loss;
• Postoperative hospital stay;
• Duration of chest tube placement;
• Length of hospitalization;
• Incidence of postoperative complications, which mainly include pneumonia, arrhythmia, incision infection, vocal cord paralysis, and trachea cannula;
• Conversion rate, defined as the rate of conversion to open surgery in the operation;
• Operative accident event, defined as the accident event that happened in operative, for example, a segmentectomy is converted to a lobectomy;
• Postoperative 30-day mortality;
• Dissection of lymph nodes, which includes overall lymph node count, number of stations dissected, and number of lymph nodes in each lymph node station;
• Five-year overall survival (OS) rate;
• Five-year disease-free survival rate;
• Preoperative lung function, which includes forced expiratory volume at 1 s (FEV1) in liter and maximal voluntary ventilation (MVV) in liter; and
• Postoperative lung function, which includes FEV1 in liter and MVV in liter;

Other prespecified outcome measures include total hospitalization expenditures and anatomical variations. Anatomical variations are defined as the rates of different anatomical variations of segmental bronchus and pulmonary vessels in the included patients.

### Randomization

Stratified blocked randomization was used for the randomization of the eligible patients. The randomization sequence was generated by using the Statistical Analysis System software version 9.4 (SAS Institute Inc., Cary, United States). Randomization was stratified by the surgeons. For each surgeon, a unique randomization sequence was generated by using the random permuted-block design (with blocks of varying sizes) to randomize patients in a 1:1 ratio to one of the two groups, 3D reconstruction CT or the standard chest CT. This method ensures that an approximately equal number of patients will be allocated to each treatment group.

### Treatment methods

**Figure 1:**
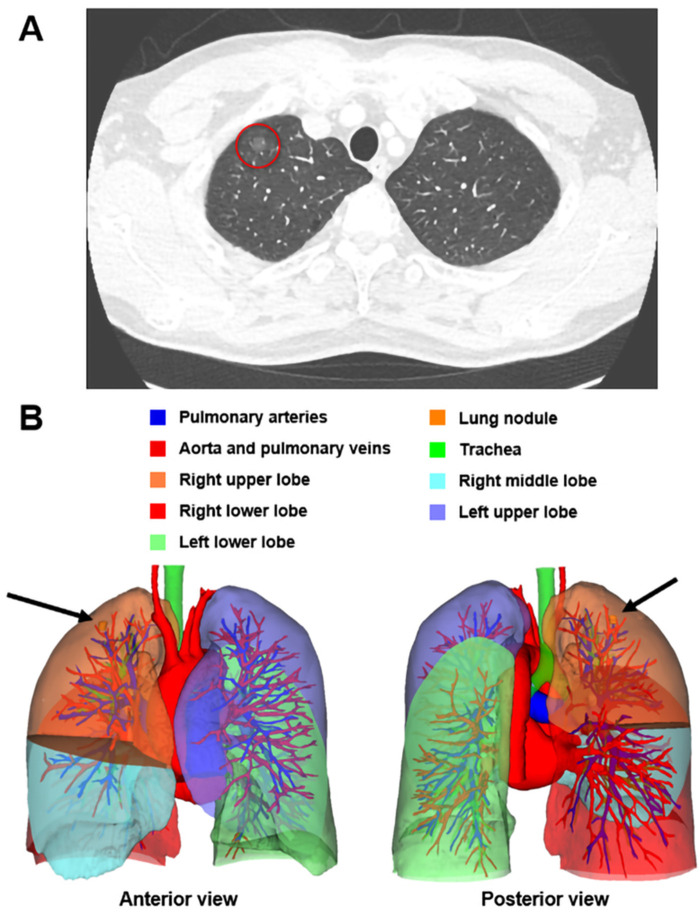
A 47-year-old male patient was admitted into our hospital after his chest CT scan revealed a GGO (**A**, red circle) in his right lung in his routine physical examination. The preoperative 3D reconstruction CT located the nodule in the peripheral region of the apical segment of the right upper lobe, measuring 7.6 mm × 7.3 mm × 7.8 mm in size (**B**, black arrow). This man received an apical segmentectomy and the pathology of this nodule was AIS. AIS, adenocarcinoma *in situ*; CT, computed tomography; GGO, ground-glass opacity; 3D, three-dimensional.

Three-dimensional reconstruction CT and standard chest CT were conducted preoperatively to assess the location of the lung nodule. In both groups, contrast-enhanced CT was performed in the supine position and full limb extension with 1 mm slice thickness. In the 3D reconstruction CT group, contrast-enhanced CT imaging data were imported into a 3D image-processing software for the 3D reconstruction of the pulmonary lobes, vessels, and bronchial trees. An example of the 3D reconstruction CT is illustrated in Figure 1.

In both groups, video-assisted thoracoscopic segmentectomy is performed with double-lumen endotracheal intubation and combined intravenous and inhalation anesthesia. The location and number of thoracoscopic ports are not limited, which depend on the habits of the surgeons. Surgical procedures include segmental resection of the lesion and mediastinal lymph node sampling (subsegmental resection or combined subsegmental resection are not included in this study). The distance from the dissection margin to the tumor edge must be no less than the maximum tumor diameter.

Appropriate N1 and N2 lymph node stations should be dissected for each patient. Hilar lymph node dissection must include ipsilateral node dissection of stations 10–13. The lymphatic drainage not only reaches the lymph nodes of the resected segment but also reaches the lymph nodes posterior to the resected segment (24). Therefore, the posterior node station 13 should be dissected, particularly when the tumor is located in the anterior segments. Node stations 12 or 13 are required for intraoperative frozen section analysis. For mediastinal lymph nodes sampling, the dissected N2 lymph nodes stations are dependent on the location of the lung nodule, based on the lobe-specific lymphatic drainage pattern (25). The mediastinal lymph node sampling will be carried out irrespective of the status of N1 lymph nodes.

### Follow-up

All randomized patients are followed up for 5 years. Standard chest CT is evaluated at least every 6 months during the first 2 years and at least every 12 months for the duration of follow-up. When local recurrence or distance metastasis is suspected during follow-up, an additional visit will be scheduled, and contrast-enhanced chest CT or PET-CT will be performed.

### Statistical analysis

According to Yao et al. (26), the operative time of segmentectomy with 3D image reconstruction was reported to be 121.5 min (range, 95–210 min). A retrospective study comparing 3D reconstruction CT and standard chest CT for segmentectomy reported the operative time to be 141.9 ± 29.1 and 160.9 ± 31.5 min, respectively (22). In our clinical center, we summarized the operative time of 124 cases of video-assisted segmentectomy with standard chest CT, which was 141.1 ± 40 min. Based on previous studies, the operative time of 3D reconstruction CT and standard chest CT for segmentectomy was assumed to be 120 and 140 min, respectively, with a difference of 20 min. With a power of 80%, a sample size of 86 will be required to detect a difference in operative time between 3D reconstruction CT group and standard chest CT group with a two-sided significance level of 5%. To allow for a 10% of dropout cases, the sample size was increased to 95 cases in each group. Data analysis will comply with the intention-to-treat principle. Data will be recorded and collected *via* standardized case report form.

## Discussion

**Figure 2:**
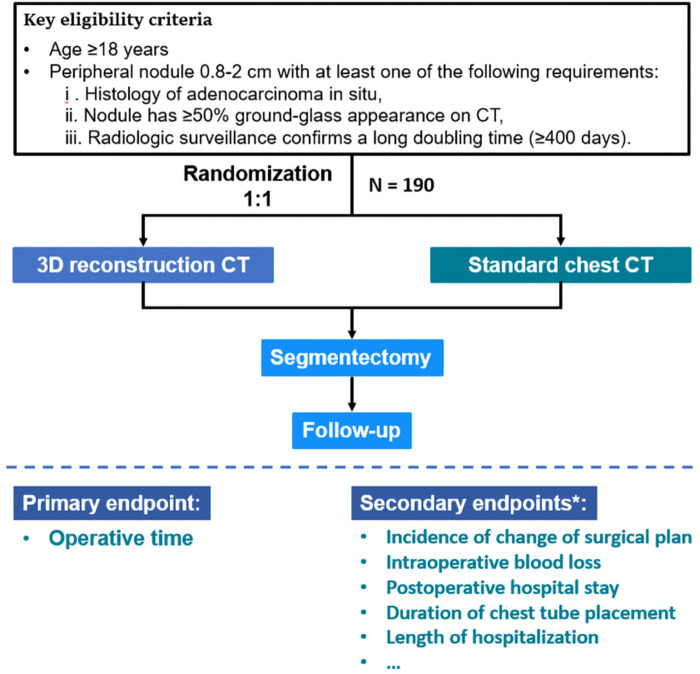
Trial design and flow chart of the DRIVATS study. *Details of the secondary endpoints are described in Materials and methods. CT, computed tomography; 3D, three-dimensional.

The DRIVATS study is a prospective, multicenter, randomized controlled clinical trial that aims to compare the usefulness of 3D reconstruction CT and standard chest CT in video-assisted segmentectomy. The flowchart of this study is shown in Figure 2. By conducting this clinical trial, we aim to provide evidence of the use of 3D reconstruction CT in the preoperative planning of lung segmentectomy.

Pulmonary segmentectomy was first described in 1,939 for the bronchiectasis of lingula segment in the left upper lobe (27). Later, the feasibility of lung segmentectomy in the treatment of lung cancer was further investigated in several studies, which showed comparable results compared with lobectomy (28–30). In 1995, as the landmark clinical trial by the Lung Cancer Study Group (31) showed a higher death rate and local recurrence rate associated with limited resection, lobectomy was then considered the standard surgical procedure of choice for patients with peripheral T1N0 NSCLC. However, as more and more smaller lung nodules (≤2.0 cm) were detected, retrospective studies from single or several institutions showed that segmentectomy for tumors ≤2.0 cm also had excellent local control, with comparable survival with lobectomy, especially when performed by VATS (32–35). Recently, with the publication of the long-awaited prospective clinical trial JCOG0802, together with JCOG0804, which confirmed the noninferior results of relapse-free survival and OS brought by segmentectomy compared with lobectomy, segmentectomy has been established as a noninferior surgical procedure compared with lobectomy for patients with peripheral tumor ≤2 cm (5, 6).

When performing a segmentectomy, it is of great importance to achieve adequate margins and complete removal of the segment containing the tumor, which is not easy since the tumor is not always palpable. Thorough knowledge of segmental anatomy and tailored preoperative planning with the assessment of surgical margins are the essential prerequisite for a successful segmentectomy (36). Chest CT plays an indispensable role in the preoperative planning of any pulmonary surgery. However, it is hard for surgeons to have a thorough understanding of lung anatomy with only conventional CT images. The introduction of 3D reconstruction CT for preoperative assessment, which allows an intuitive recognition of the anatomy, has gained popularity amongst thoracic surgeons (16, 19, 20, 37). Pulmonary artery branches can be precisely identified in 3D reconstruction CT images, with an accuracy rate of over 95% based on previous studies (12, 20, 38). Additionally, preoperative 3D CT could significantly shorten the operative time of segmentectomy and lead to reduced intraoperative blood loss and postoperative complications compared with preoperative conventional 2D CT (22, 23).

Identification of anatomical anomalies is especially helpful for segmentectomy, which may increase the risks of complications during surgery. 3D imaging can be used for screening anatomical anomalies (7–9). Based on the 3D CT images, different patterns of anatomical variations have been identified and categorized, which could facilitate the creation of simplified models for use in preoperative planning for segmentectomy (10–13). In this DRIVATS study, we also aimed to prospectively collect the anatomical anomalies in the included patients. This anatomical data, along with all possible variations, will surely facilitate safe and accurate lung resections for thoracic surgeons, irrespective of the presence of preoperative 3D reconstruction CT.

Accurate pathologic nodal staging is especially important for sublobar resections like segmentectomy. False-negative results of lymph nodes have a negative impact on the prognosis of the patients (39). Based on a large cohort of NSCLC ≤3 cm, Naruke et al. (40) reported that the rates of lymph node stations 12 and 13 metastases were 12.4% and 8.4%, respectively. When the intrapulmonary lobar and segmental lymph nodes were not dissected, the false-negative rate for N staging would be 9.0% (41). In this DRIVATS study, intraoperative fast-frozen pathology of node stations 12 and 13 is mandatory which is a key surgical procedure to ensure the oncological effect of segmentectomy. Segmentectomy would not be suitable if metastasis to the adjacent segmental lymph nodes was confirmed by fast-frozen pathology.

However, some practical and operational limitations exist when performing this multicenter clinical trial. First, the endpoints of our study may be susceptible to some confounding factors such as the surgical procedures and techniques, and the experience of the surgeons. We believe randomization and subsequent subgroup analysis may relieve this bias to some extent. Second, the different types of CT scanners as well as reconstruction techniques adopted by different medical centers may affect the study results. This may have an impact on the generalization of this study's conclusions.

In summary, the DRIVATS study is a prospective, multicenter, randomized controlled clinical trial that aims to compare the usefulness of 3D reconstruction CT and standard chest CT in video-assisted segmentectomy. The results of this study will facilitate the clinical application of segmentectomy and improve the accuracy and safety of segmentectomy.

## Data Availability

The original contributions presented in the study are included in the article/Supplementary Material, further inquiries can be directed to the corresponding authors.
